# Broadband and Multi-Cylinder-Based Triboelectric Nanogenerators for Mechanical Energy Harvesting with High Space Utilization

**DOI:** 10.3390/ma16083034

**Published:** 2023-04-12

**Authors:** Xu Chen, Bao Cao, Chao Yang, Haonan Zhang, Lin Fang, Chen Chen, Zixun Wang, Wen He, Peihong Wang

**Affiliations:** 1 Energy Materials and Devices Key Laboratory of Anhui Province for Photoelectric Conversion, School of Materials Science and Engineering, Anhui University, Hefei 230601, China; 2 Key Laboratory of Structure and Functional Regulation of Hybrid Materials, Anhui University, Ministry of Education, Hefei 230601, China

**Keywords:** triboelectric nanogenerators, broadband, high space utilization, self-powered, blue energy

## Abstract

The development and utilization of new energy sources is an effective means of addressing the limits of traditional fossil energy resources and the problem of environmental pollution. Triboelectric nanogenerators (TENG) show great potential for applications in harvesting low-frequency mechanical energy from the environment. Here, we propose a multi-cylinder-based triboelectric nanogenerator (MC-TENG) with broadband and high space utilization for harvesting mechanical energy from the environment. The structure consisted of two TENG units (TENG I and TENG II) assembled by a central shaft. Both an internal rotor and an external stator were included in each TENG unit, operating in oscillating and freestanding layer mode. On one hand, the resonant frequencies of the masses in the two TENG units were different at the maximum angle of oscillation, allowing for energy harvesting in a broadband range (2.25–4 Hz). On the other hand, the internal space of TENG II was fully utilized, and the maximum peak power of the two TENG units connected in parallel reached 23.55 mW. In contrast, the peak power density reached 31.23 Wm^−3^, significantly higher than that of a single TENG unit. In the demonstration, the MC-TENG could power 1000 LEDs, a thermometer/hygrometer, and a calculator continuously. Therefore, the MC-TENG will have excellent application in the field of blue energy harvesting in the future.

## 1. Introduction

With the development of global industries, energy demand has increased dramatically [1]. Fossil energy accounts for a significant part of energy use, but burning fossil fuels has many negative aspects such as the greenhouse effect and harmful particles [2,3]. This problem is compounded by fossil energy being non-renewable and finite. The energy crisis is becoming increasingly severe, so more attention has been given to the development and use of renewable energy sources [4,5,6]. Solar, wind, hydro, and nuclear energy are green energy sources that have long been in the public eye. However, the oceans, which cover the most significant part of the Earth’s surface, also contain massive energy [7,8,9,10]. Tidal and ocean currents have been exploited to generate electricity, but water wave energy is not still well-utilized [11,12,13,14]. It is, therefore, of great interest to develop an energy harvesting device that can efficiently harvest water wave energy and is environmentally friendly. Most modern electronics collect energy primarily by electromagnetic induction [15,16]. Nevertheless, their primary drawback is that they require sophisticated procedures such as manual charging and maintenance, particularly at low-frequencies where energy conversion is less efficient [17,18,19].

In 2012, a novel energy conversion technology, called a triboelectric nanogenerator, which was first proposed by Prof. Z. L. Wang, attracted widespread attention, as its main purpose is to collect low amounts of mechanical energy [20,21,22,23]. Because of the advantages of small size, low cost, and high energy conversion efficiency, TENG has great advantages in the field of energy harvesting and self-powered sensing [24]. Triboelectric nanogenerators are based on the coupling effects of triboelectrification and electrostatic induction [25]. They use two triboelectric materials with different polarities rubbing against each other to produce two charges of opposite polarity on their respective surfaces. Due to its own unique working mechanism, the triboelectric nanogenerator is significantly better than the conventional electromagnetic generator in collecting low-frequency energy, especially the water wave energy in oceans [26,27,28]. Currently, the pendulum type P-TENG proposed by Lin et al. has good electrical output performance around 2 Hz, but a large part of the space inside its spherical shell is wasted [29]. Similarly, Rui et al. reported a cylindrical pendulum shaped triboelectric nanogenerator (CP-TENG) whose main structure consisted of two cylinders assembled to collect the wave energy inside the ocean by the internal rotor oscillation. However, the disadvantage of this structure is that there is still a great deal of unused space inside, and the efficiency of space utilization could be higher. Since the structure has good output performance in a minimal frequency range and almost stops working when the oscillation frequency is far from the optimal frequency, the structure cannot adapt to the complex and changing marine environment [30].

In this paper, we proposed a multi-cylinder-based triboelectric nanogenerator with broadband and high space utilization to address the problems of narrowband range and low space utilization in the previously reported work. The two TENG units contained in the device both consisted of a rotor and a stator using arched FEP films and aluminum electrodes as the friction materials. The design of the multi-cylinder structure improved the utilization of internal space and electrical output performance. On the other hand, the characteristic of different oscillating parts with different resonant frequencies was used to achieve broadband energy harvesting. First, the effect of the perimeter and thickness of the arched FEP films on the electrical output performance of each MC-TENG unit was investigated. Next, the mass size of the mass block and the motion amplitude of the linear motor was adjusted to find the optimal resonant frequency of the two TENG units, in addition to illustrating the ability of the TENG to generate energy harvesting in multiple directions. Finally, the ability of MC-TENG to power miniature electrical devices was demonstrated. It was further demonstrated that MC-TENG is essential for future mechanical energy harvesting in the environment and for powering microelectronic equipment.

## 2. Experimental Section

### 2.1. Fabrication of the MC-TENG

The MC-TENG consists of two central TENG units (TENG I and TENG II). Each TENG unit consisted of two acrylic cylinders forming the inner rotor and outer stator. In TENG I, the rotor (outer diameter: 50 mm, inner diameter: 46 mm, height: 70 mm) had 15 FEP arched films (circumference: 20 mm, thickness: 30 μm, length: 66 mm) attached to the outer wall and lead strips (mass: 60 g) attached to the inner wall of the rotor; the stator (outer diameter: 65 mm, inner diameter: 61 mm, height: 78 mm) was laminated with a PET film on the inner wall and an aluminum foil (length: 76 mm, width: 5 mm) on top of the PET film, where the aluminum foil had a complementary pattern and a gap of 1 mm, with twice as many aluminum electrodes as there were FEP arches. In TENG II, 15 FEP arched films (perimeter: 28 mm, thickness: 30 um, length: 90 mm) were attached to the outer wall of the rotor (outer diameter: 90 mm, inner diameter: 86 mm, height: 90 mm), a strip of lead (mass: 210 g) was attached to the inner wall of the rotor, and a ring of PET film was attached to the inner wall of the stator (outer diameter: 105 mm, inner diameter: 99 mm, height: 98 mm). The aluminum foil (length: 96 mm, width: 10 mm) had a complementary pattern and a gap of 2 mm, with twice as many aluminum electrodes as the FEP arches. The bearings were embedded in the middle of the cover plates at both ends of the rotor, which were then fixed to the central shaft for easy oscillation; the stator’s upper and lower cover plates were fixed directly to the central shaft. The wires of the TENG I section were threaded through the hollow part of the central acrylic shaft, and the MC-TENG unit was completely sealed to ensure a stable output.

### 2.2. Electrical Measurements of the MC-TENG

The electrical output performance of the CP-TENG was measured by an electrostatic meter (6514, Keithley, Cleveland, OH, USA). A data acquisition card (NI, USB 6211, Austin, TX, USA) and the LabView software platform were used to acquire and analyze the data. A commercial motor (ZD-5IK40W, Ningbo, China) was used to drive the TENG. The mass block’s deflection angle was measured with the Vettel Smart 6-axis Bluetooth attitude gyro sensor (BWT61CL).

## 3. Results and Discussion

### 3.1. Structure and Working Principle of the MC-TENG

**Figure 1:**
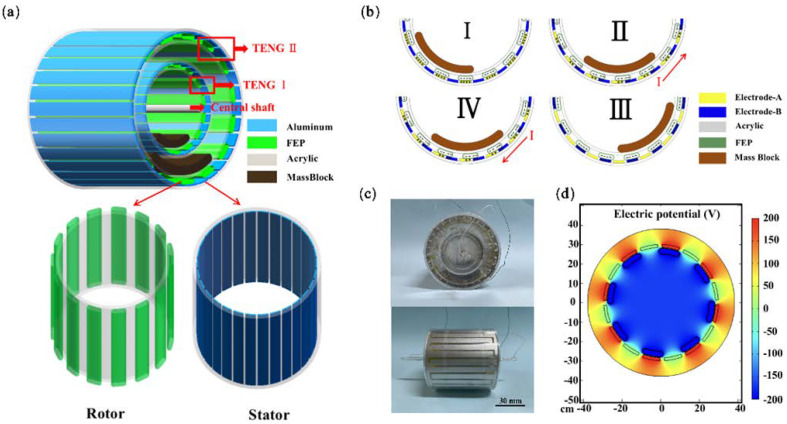
Schematic diagram of the structure and working principle of the sleeve-type TENG. (**a**) The structural design of the MC-TENG including the internal rotor and the external stator. (**b**) Charge distribution during the MC-TENG rotor oscillation. (**I**–**IV**) (**c**) Photograph of the MC-TENG. (**d**) The potential distribution of MC-TENG was calculated by the COMSOL simulation.

The structure diagram of MC-TENG and the working schematic are shown in Figure 1. The MC-TENG consisted of two TENG units (Figure 1a), where each TENG unit consisted of an internal rotor and an external stator. TENG I mainly collects high-frequency mechanical vibration energy, and TENG II collects low-frequency mechanical vibration energy. The difference between the two TENG units is in their sizes. Two acrylic hollow cylinders were snapped together to form the rotor and stator of each TENG unit. Fifteen arched FEP films were attached to the external surface of the rotor, and lead wires were attached to the internal surface as mass blocks. Thirty interdigitated electrodes were affixed to the internal surface of the stator. The acrylic center shaft was fixed to the stator cover after passing through the bearing in the middle of the rotor cover, which allows the rotor to move relative to the stator when it oscillates. Since the contact between the arched FEP film and the electrode is soft contact, compared with the point contact or wire contact form of the previous TENG, the friction is significantly reduced [31,32,33]. The contact area between the arched FEP films and the aluminum electrode was larger, and the electrical output performance of the TENG was better. In addition, the arched FEP films allowed the rotor to rotate back and forth in both the clockwise and counterclockwise directions during operation, improving the energy harvesting efficiency. The operation of a TENG unit in the freestanding layer mode is shown in Figure 1b. After soft contact, the arched FEP film slides relative to the aluminum electrode, and the negative charge will be concentrated in the arched FEP film. Meanwhile, the positive charge will be concentrated in the aluminum electrode part due to the position of the aluminum ahead of the FEP in the frictional electric sequence [30,34].

As shown in Figure 1(bI), in the initial state, if both the FEP and aluminum electrodes are not charged after soft contact, an electrostatic charge is generated due to triboelectrification. As the arched FEP film is in complete contact with aluminum electrode A, the negative charge on the surface of the FEP membrane and the positive charge on the surface of the aluminum electrode A should be equal and there is no current generation in the external circuit due to the charge balance. In Figure 1(bII), when TENG I is subjected to the external vibration energy, the mass block on the inner wall of the rotor swings to the right, and the arched FEP film is partially in contact with electrode B. Currently, the charge of electrode A flows into electrode B, while a counterclockwise current is generated in the external circuit. In Figure 1(bIII), as the mass block continues to swing so that the arched FEP films are in complete contact with electrode B, the negative charge on the arched FEP films is wholly neutralized with the positive charge on electrode B. In Figure 1(bIV), the mass block reaches the highest position and then swings to the left, making the arched FEP film come into contact with electrode A again [35]. The direction of the external current changes, and finally, the arched FEP films again returns to its initial position. Figure 1(bI–IV) describes the complete working process of a TENG unit. The mass block’s periodic swinging back and forth generates an alternating current in the external circuit. The potential distribution in two electrodes in a TENG unit is simulated by the COMSOL finite element method, as shown in Figure 1c, which can explain the principle of the current generation in the external circuit more clearly.

### 3.2. Performance of the MC-TENG

**Figure 2:**
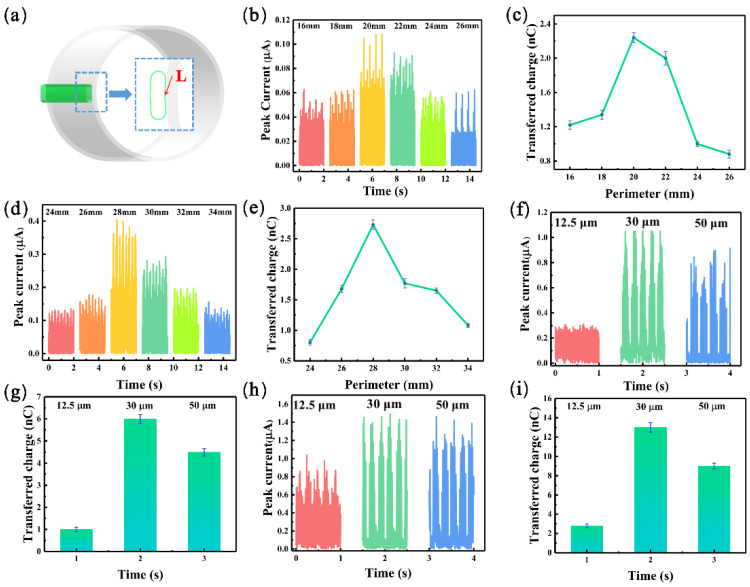
The effect of different parameters of the arched FEP film on the electrical output performance of MC-TENG. (**a**) Adjustment of the perimeter and thickness of the films. (**b**–**e**) Variation in the peak current and transferred charge for different perimeters of FEP arches in each TENG unit. (**f**–**i**) The peak current and the amount of transferred charge for different thicknesses of FEP arches in each TENG unit.

As shown in Figure 2a, only one arched FEP film was applied to the rotor surface to save material and simplify the procedure. By adjusting the perimeter (*L*) and thickness (*H*) of the arched FEP films, the film parameters best suited for the operation of the TENG unit were optimized. In the first experiment, the effect of the perimeter of the arched FEP films on the electrical output performance of MC-TENG was investigated by fixing the thickness of the arched FEP films to 12.5 μm and then varying the perimeter of the arched FEP films in two TENG units (TENG I: 16–26 mm, TENG II: 24–34 mm). Figure 2b–e shows the peak currents and transferred charges of TENG I and TENG II at different arched FEP film perimeters. At an *L* value of 20 mm, the maximum peak current in the TENG I unit was 0.11 μA, and the corresponding transfer charge was 2.24 nC. In contrast, the TENG II unit could generate a maximum peak current of 0.41 μA and a transfer charge of 2.73 nC at an L value of 28 mm. The electrical output performance of the TENG decreased when the perimeter of the arched FEP films exceeded the optimum *L* value because the spacing between the rotor and the stator was limited. The perimeter of the arched FEP films will contact the two adjacent electrodes simultaneously when the perimeter of the arched FEP films is too large, and the output performance is negatively affected. Therefore, the *L* value of TENG I was determined to be 20 mm and that of TENG II to be 28 mm. 

We continued to investigate the effect of the thickness of the arched FEP film on the electrical output of MC-TENG. Figure 2f,g shows the output performance of TENG I under different thicknesses of arched FEP film, and it was found that the maximum peak current value was 1.1 μA, and the transferred charge was 6 nC at *H* = 30 μm. As shown in Figure 2h,i, TENG II had a maximum peak current value of 1.5 μA and a transfer charge of 13 nC at *H* = 30 μm. At 12.5 μm, the thickness of the arched FEP films was small, and the repulsion of the electrostatic force resulted in insufficient contact between the arched FEP films and the aluminum electrode. The output performance of TENG was negatively affected. The thickness of the arched FEP film was larger than 50 μm, and the hardness was more prominent after the roll, which increased the frictional resistance during contact with the electrode, thus accelerating the wear of the device. Therefore, the thickness of the FEP arch film in TENG was set as 30 μm. The above data were acquired on a linear motor with excitation parameters of *A* = 30 mm, *f* = 2 Hz, and *m* = 120 g. Since only one arched FEP film was applied and the frictional resistance between the rotor and the stator was low, the excitation parameters were chosen to ensure that the rotor oscillated periodically and that the arched FEP films could cross one electrode. 

**Figure 3:**
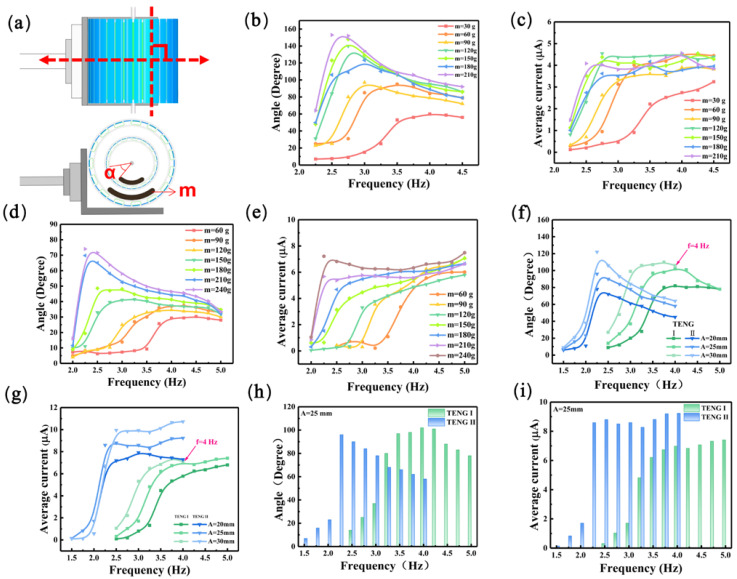
The effect of the mass block and excitation amplitude on the output of MC-TENG. (**a**) Relative position of the MC-TENG to the linear motor. (**b**–**e**) Variation in the mass block mass in each TENG unit concerning its swing angle and the average TENG current value. (**f**,**g**) The effect of different excitation amplitude conditions on the swing angle of the mass block in the two TENG units and the average current value. (**h**,**i**) Comparison of the outputs of the two TENG units at different frequencies.

Next, we continued to study the effects of different external conditions on the electrical output performance of the MC-TENG. The direction of motion of the linear motor in Figure 1a was ensured to be perpendicular to the central shaft. The mass block’s mass (*m*) inside the MC-TENG rotor and the linear motor’s motion frequency (*f*) were varied. The swing angle (*α*) of the mass block was measured using an angle sensor, and the average short-circuit current (*I_asc_*) value in each TENG unit cycle was also calculated. The mass block was chosen between 30 g and 210 g for TENG I and 60–240 g for TENG II. As in Figure 3b,d, the oscillation angle (*α*) of the mass block increased with the increase in the mass block, but the frequency (*f*) corresponding to the maximum angle of each mass block appeared to decrease. The increase in frequency led to a shortening of the periodical oscillation time of the mass block. Therefore, the resonant frequency (*f_r_*) corresponding to the maximum oscillation angle of TENG was lower for the larger mass block. In this work, we hoped that TENG I had the maximum swing angle under high frequency vibration, while TENG II had the maximum swing angle under low frequency vibration. The maximum swing angle corresponded to the best resonant frequency (*f_r_*) of each TENG unit, and the purpose was to make the MC-TENG meet the broadband operating performance. As shown in Figure 3b, the maximum swing angle *α* = 60° corresponded to the mass block *m* = 30 g and the maximum swing angle α = 96° corresponded to *m* = 60 g for TENG I near *f* = 3.75 Hz, which was about 1.5 times that of the former. The *I_asc_* value after *m* = 60 g in Figure 3c tended to be stable, so we chose *m* = 60 g for the mass block of TENG I. The mass block of TENG II in Figure 3d had a mass of *m* = 210 g, and the corresponding maximum oscillation angle *α* = 70° occurred close to *f* = 2.25 Hz. However, when *f* = 2 Hz, the mass block hardly oscillated, the arched FEP films could not cross a whole electrode, and the TENG II unit had almost no electrical output at this time. As shown in Figure 3e, the corresponding *I_asc_* values stabilized after the swing angle *α* reached its maximum value for different mass blocks in TENG II. Considering the limited volume of MC-TENG, the mass block of TENG II was determined as *m* = 210 g. The excitation amplitude (*A*) of the linear motor motion in the above work was fixed as *A* = 20 mm.

After determining the mass block parameters of each unit in the MC-TENG, the continued optimization of the operating conditions was investigated by varying the linear motor of the excitation amplitude *A*. Three parameters of *A* = 20 mm, 25 mm, and 30 mm were used in the following experiments. As shown in Figure 3f,g, the deflection angle *α* and *I_asc_* of each unit’s mass block in MC-TENG increased with the excitation amplitude *A*. When *A* = 30 mm, the motion of the mass block in TENG I changed from oscillation to irregular rotation after *f* = 4 Hz because of the excessive excitation energy. Therefore, the output performance of TENG I after *f* = 4 Hz is not further discussed. Therefore, we later chose *A* = 25 m as the excitation amplitude of the linear motor. Figure 3h,i mainly shows the relationship between the mass block swing angle *α* and *I_asc_* of each TENG unit in MC-TENG and the external excitation frequency when *A* = 25 mm. The maximum swing angle *α* = 102° of the mass block in TENG I corresponded to the optimum resonant frequency (*f_r_*) size of 4 Hz. In contrast, the maximum swing angle *α* = 96° of TENG II corresponded to the optimum resonant frequency (*f_r_*) size of 2.25 Hz. 

In Rui’s work [29], the CP-TENG had relatively good electrical output, which was only around *f_r_* = 1.75 Hz. In contrast, in this work, through reasonable optimized parameters (mass block m and excitation amplitude *A*), the two TENG units in the MC-TENG could ensure that at least one TENG unit was working after *f* = 2.25 Hz. TENG II had the best output when *f_r_* = 2.25 Hz, and TENG I had the best output when its *f_r_* was equal to 4 Hz, and both TENG units could work simultaneously in the frequency interval above-mentioned. Therefore, the goal of collecting a broadband energy range was achieved.

**Figure 4:**
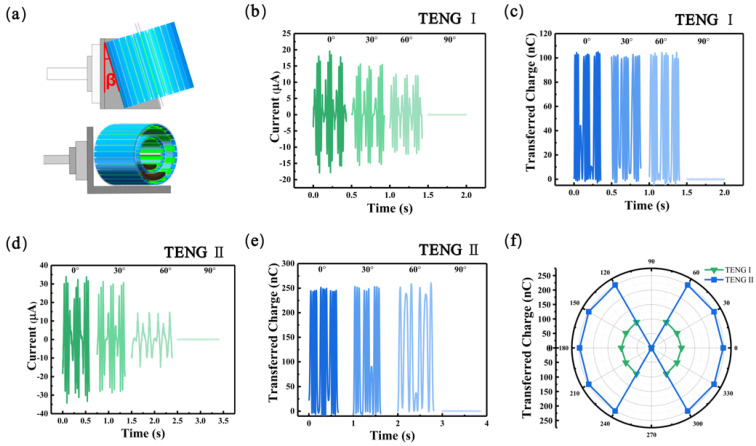
Electrical output performance of MC-TENG with different excitation directions. (**a**) The fixed position of MC-TENG on the linear motor. (**b**,**c**) The short-circuit current values and the corresponding transferred charges for different deflection angles of TENG I with a fixed amplitude of *A* = 25 mm and *f* = 4 Hz. (**d**,**e**) The short-circuit current values and the corresponding transferred charge at different deflection angles of TENG II with fixed amplitude of *A* = 25 mm and *f* = 2.25 Hz. (**f**) Transfer charge simulated by TENG from different angles.

Figure 4 demonstrates the performance of MC-TENG in multi-directional energy harvesting. In Figure 4a, we measured the short-circuit current (*I_sc_*) and transferred charge (*Q_sc_*) of each TENG unit of MC-TENG by adjusting the deflection angle (*β*) of the linear motor movement direction concerning the center shaft of MC-TENG. With *f* = 4 Hz as the TENG I excitation frequency and *f* = 2.25 Hz as the TENG II excitation frequency, the oscillation angle *α* of the mass block in each unit was the maximum at *β* = 0°. It was found in Figure 4b–e that as the angle *β* increased, the short-circuit current generated by each TENG unit gradually decreased, and the transferred charge tended to be stable. The arched FEP films could always cross a whole electrode when *β* was less than 60°, resulting in a constant amount of transferred charge. At *β* = 90°, the vibration direction of the linear motor was parallel to the center shaft of the MC-TENG, and the mass block basically did not oscillate, so there was almost no electrical output. Figure 4f shows the effective electrical output range of the MC-TENG, which could still produce a sound output even if *β* exceeded 240°. This indicates that MC-TENG can also be applied to harvest multi-directional vibration energy in the future [30,36].

### 3.3. Demonstration

**Figure 5:**
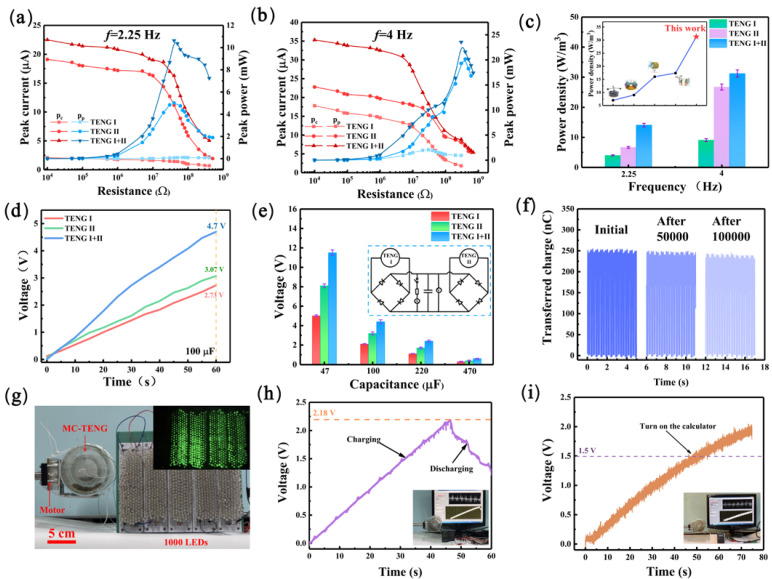
The electrical output performance and demonstration of the MC-TENG driven by a linear motor. (**a**,**b**) Output current, peak power of the MC-TENG with resistance driven by a linear motor. (**c**) The power density of the MC-TENG at different frequencies. (**d**) Time comparison of the MC-TENG to charge a 100 µF capacitor. (**e**) The charging of different capacitors by the MC-TENG. (**f**) The amount of transferred charge after 100,000 cycles. (**g**) Photograph of the MC-TENG powering 1000 LEDs. Demonstration of the MC-TENG powering a temperature/humidity meter (**h**) and a calculator (**i**).

The output power as well as the charging capacity of the MC-TENG were finally measured in this work. The peak current (*I_max_*) [36] and peak power (*P_p_*) of the respective units in the MC-TENG were first measured at low-frequency (*f* = 2.25 Hz) and high-frequency (*f* = 4 Hz) conditions under different resistances, respectively. The peak power was calculated by using the formulation of *P_p_ = I_max_*^2^*R*. The output power of the two TENG units, which were rectified and then connected in parallel, was also measured and compared with the output power of the single TENG unit. The *I_max_* decreased with increasing load resistance, while the *P_p_* increased with increasing load resistance. As shown in Figure 5a, the swing angle (*α*) of the mass block in TENG I under low-frequency conditions was slight, so there was only a 0.115 mW maximum *P_p_* at a load resistance of 70 MΩ. The maximum *P_p_* in TENG II was 5.02 mW at 40 MΩ. The maximum *P_p_* of the two TENG units connected in parallel was 10.62 mW at 40 MΩ, roughly twice the sum of the power values of the two independent TENG units. In Figure 5b, the *P_p_* of TENG I at 30 MΩ was 2.07 mW. The maximum *P_p_* of TENG II at 20.16 mW occurred at 4 × 10^2^ MΩ. Moreover, when the load resistance was 3 × 10^2^ MΩ, the *P_p_* of the TENG after its parallel connection reached 23.55 mW. The volume of TENG I was 2.278 × 10^−4^ m^3^, and the volume of TENG II was 7.54 × 10^−4^ m^3^, so the power density of each TENG unit corresponding to the peak power under different conditions could be calculated. As shown in Figure 5c, the power density of TENG I was 0.05 Wm^−3^ and 9.07 Wm^−3^, and the power density of TENG II was 6.66 Wm^−3^ and 26.74 Wm^−3^ at low frequency (*f* = 2.25 Hz) and high frequency (*f* = 4 Hz), respectively. The power density of the TENG units connected in parallel was 14.08 Wm^−3^ and 31.23 Wm^−3^, respectively. The power density of MC-TENG was significantly better than that in the previous work [37,38,39,40]. After rectification, the power density of the TENG units connected in parallel was significantly higher than that of a single TENG unit, which proves that the MC-TENG makes full use of the space inside the cylinder and improves the space utilization to a large extent [29].

To study the charging capability of the MC-TENG, a 100 μF capacitor was connected to the circuit of MC-TENG. Since the output of TENG was poor at low-frequency and the peak power of MC-TENG was more significant at high-frequency, high-frequency (*f* = 4 Hz) was chosen to observe the charging capability of the MC-TENG. As shown in Figure 5d, after 60 s, TENG I charged the 100 μF capacitor to 2.73 V, TENG II could charge it to 3.07 V, and the 100 μF capacitor could be charged to 4.7 V by the two TENG units after rectification. To demonstrate the charging capability more visually, several other parameters of the capacitors were chosen to be connected in the MC-TENG. As shown in Figure 5e, the larger the capacitor value, the longer the charging time. TENG II was tested for endurance because the device is subject to wear and tear. As shown in Figure 5f, the transfer charge of the TENG II could still be maintained at about 250 nC through 100,000 cycles of continuous operation, and the transfer charge decayed less with an increasing cycle time. This output performance of MC-TENG could be used to drive some miniature electronic devices. Figure 5g and Supplementary Video S1 show that the MC-TENG can continuously light up 1000 LEDs. Figure 5h and Supplementary Video S2 used the MC-TENG to power a temperature/humidity meter for some time when the capacitor was charged to 2.18 V. Figure 5i and Supplementary Video S3 show that the calculator can work continuously after charging to 1.5 V. It is worth noting that the charging curve kept rising. However, the curve’s slope decreased slightly, indicating that the harvested vibration energy can ensure the continuous operation of the calculator and the meter. These experiments allow for the future application of the TENG in the ocean, where the waves must lap, causing the mass block of the MC-TENG to oscillate, thus generating an electrical output to power some miniature electronic devices.

## 4. Conclusions

In summary, this work designed a multi-cylinder-based triboelectric nanogenerator with broadband and high space utilization based on the conventional oscillating triboelectric nanogenerator. The MC-TENG mainly consisted of two TENG units, each unit containing a pair of an internal rotor and external stator. The relative sliding between the rotor and the stator, driven by the external energy, forms a TENG in the freestanding layer mode. The difference in the size of the two TENG units and the mass block masses makes the maximum angle of mass block oscillation in each TENG unit correspond to different resonant frequencies. The resonant frequency of TENG I was *f_r_* = 4 Hz, which is a relatively high frequency, while the resonant frequency of TENG II was *f_r_* = 2.25 Hz, which is a relatively low frequency. Therefore, MC-TENG can collect mechanical energy in a complex external environment with a broad frequency range. The maximum power density of two TENG units connected in parallel could reach 31.23 Wm^−3^, making full use of the device’s internal space and significantly improving the space utilization. The MC-TENG can also collect energy in over 240° directions, offering the advantage of multi-directional energy harvesting. The demonstration illustrates that MC-TENG can power 1000 LEDs continuously and power a temperature/humidity meter and a calculator that work intermittently. 

## Data Availability

Not applicable.

## Supplementary Materials

The following supporting information can be downloaded at: https://www.mdpi.com/article/10.3390/ma16083034/s1, Video S1: The MC-TENG can continuously light up 1000 LED; Video S2: The MC-TENG to power a temperature/humidity meter; Video S3: The MC-TENG to power a calculator.
